# The interferon induced with helicase domain 1 A946T polymorphism is not associated with rheumatoid arthritis

**DOI:** 10.1186/ar2179

**Published:** 2007-04-18

**Authors:** Ioanna Marinou, Douglas S Montgomery, Marion C Dickson, Michael H Binks, David J Moore, Deborah E Bax, Anthony G Wilson

**Affiliations:** 1 Section of Musculoskeletal Sciences, School of Medicine & Biomedical Sciences, The University of Sheffield, Royal Hallamshire Hospital, Sheffield S10 2RX, UK; 2 GlaxoSmithKline R&D, Stevenage SG1 2NY, UK

## Abstract

An important feature of autoimmune diseases is the overlap of pathophysiological characteristics. Clustering of autoimmune diseases in families suggests that genetic variants may contribute to autoimmunity. The aim of the present study was to investigate the role of the interferon induced with helicase domain 1 (IFIH1) A946T (rs1990760 A>G) variant in rheumatoid arthritis (RA), as this was recently associated with susceptibility to type 1 diabetes. A total of 965 Caucasians with RA and 988 healthy controls were genotyped for IFIH1 A946T. Gene expression of IFIH1 was measured in peripheral blood leukocytes using real-time PCR. Genotypes were equally distributed in both RA cases and healthy controls (odds ratio for allele C = 0.9, 95% confidence interval = 0.8–1.0, *P *= 0.3). No association was detected after stratification by sex, age at onset, rheumatoid factor status, anti-cyclic citrullinated peptide status or radiological joint damage. Levels of IFIH1 mRNA were approximately twofold higher in blood leucocytes of RA cases compared with healthy controls (*P *< 0.0001). These results indicate that the IFIH1 is upregulated in RA but that the A946T variant does not contribute significantly to the genetic background of RA.

## Introduction

Autoimmune diseases are characterised by a loss of immunological tolerance and by chronic inflammation that frequently results in tissue damage. Collectively the diseases affect about 4–5% of the population and have a multifactorial origin involving both genetic and environmental factors [1]. An important feature of autoimmune diseases is the overlap of common pathophysiological characteristics as well as their co-occurrence in families, as indicated by studies documenting the increased prevalence of rheumatoid arthritis (RA) and other autoimmune diseases including type 1 diabetes (T1D) [2,3]. Such findings suggest the presence of common genetic variants predisposing to autoimmunity.

The region most commonly associated with susceptibility to autoimmune diseases is the human leukocyte antigen class II genes on chromosome 6p21.3 [4-6]. Approximately one-third of the genetically encoded risk of developing RA arises from DRB1 alleles, although recent evidence suggested that this association is primarily with production of anti-cyclic citrullinated peptide (anti-CCP) antibodies [7]. The identification of disease-related genes outside the major histocompatibility complex has been much more difficult; however, recent successes have included the identification of cytotoxic-T-lymphocyte-associated 4 with Graves disease and T1D [8,9], and, more recently, the association of PTPN22 with several diseases including T1D [10], Graves disease [11], RA [12] and systemic lupus erythematosus [13].

The interferon induced with helicase C domain 1 (IFIH1) gene, also referred to as *mda-5 *or *Helicard*, is located at 2q24.3 and encodes an early type 1 IFN response gene. It is a helicase that detects dsRNA, resulting in the activation of transcription factors such as IFN-regulatory factor 3 and NF-κB. IFIH1 is expressed at low levels in most tissues, with relatively higher expression in immune cells. A recent study reported convincing statistical evidence for IFIH1 being the sixth identified T1D susceptibility locus with association of a nonsynonomous single nucleotide polymorphism, A946T (rs1990760 A>G) [14]. The A946T SNP leads to an alanine for threonine substitution at position 946 and the C allele was found to be a risk factor for T1D. To determine whether this variant is involved in the genetic background of RA, we compared genotypes in a large case–control study and also in relation to clinical features including rheumatoid factors (RFs) and anti-CCP status and radiological joint damage. We examined levels of IFIH1 mRNA in peripheral blood leukocytes of patients and controls.

## Materials and methods

### Study populations

A total of 965 white Caucasian individuals with RA and of 988 healthy unrelated individuals participated in this study. The control group was from the Sheffield area and each individual was 18 years of age or older with no history of an inflammatory joint disorder. The South Sheffield Research Ethics Committee approved this study and informed consent was obtained from all participants. RA was diagnosed according to the American College of Rheumatology diagnostic criteria. The measurements of RFs, anti-CCP and modified Larsen scores were obtained as previously described [7].

### Single nucleotide polymorphism genotyping

Blood samples were collected in ethylenediamine tetraacetic acid-anticoagulated tubes and DNA was extracted using standard methods. A Taqman SNP genotyping assay was designed for rs1990760 by Applied Biosystems (PE Biosystems, Foster City, CA, USA). The sequences of the primers were, 5'-ACCATTTATTTGATAGTCGGCACACT-3' (forward) and 5'-CCCTTTGATACTTATAGGGAACTTTACATTGT-3' (reverse). The sequences of the allele-specific probes were 5'-TTTTGCAGTGCTTTGTT-3' for the C allele (reporter dye VIC) and 5'-CTTTTGCAGTGTTTTGTT-3' for the T allele (reporter dye FAM.

Thermal cycling was performed as follows; after an initial denaturation and enzyme activation of 10 minutes at 95°C, samples were subjected to 40 cycles of 15 seconds at 95°C for denaturation and 60 seconds at 60°C for annealing/extension. To ensure the accuracy of genotyping results we included multiple positive and negative controls in all genotyping plates, and we repeated 10% of our samples to eliminate genotyping errors. Thermal cycling in 384-well plates was performed on the PTC-225 DNA engine Tetrad (MJ Research, San Francisco, CA, USA) and genotypes were determined using an ABI Prism 7900 HT (PE Biosystems). Genotyping was confirmed by DNA sequencing of the six A946T individuals, comprising two of each genotype.

### mRNA quantitation

Whole blood was collected into Qiagen PAXgene tubes and total RNA was extracted according to the PAXgene RNA system (Qiagen, Crawley, UK). The synthesis of cDNA and the gene expression analysis were performed in 200 RA cases and in 200 healthy controls as described previously [15]. The sequences of the primers were 5'-CAGTGTGCTAGCCTGTTC-3' (sense) and 5'-TCCTTGAATTCTGGGGTC-3' (antisense). The levels of IFIH1 mRNA in each sample were normalised using the housekeeping gene GAPDH as described previously [15].

### Statistical analysis

The Hardy–Weinberg equilibrium was tested separately in cases and controls using a chi-square test. A threshold of *P *< 0.05 was used to indicate departure from Hardy–Weinberg equilibrium. Association with susceptibility to RA was analysed by applying a chi-square test on 2 × 2 contingency tables. The odds ratios were calculated with the 95% confidence interval, and *P *< 0.05 was considered significant. Association with clinical features such as sex, age at onset, X-ray damage (as defined by the modified Larsen score), RF status and CCP status with each genotype were then analysed using a chi-square test, the Kruskal–Wallis test or Cuzick's trend test as appropriate. A cutoff value of 40 IU/ml and a cutoff value of 5.5 U/ml were used as a criterion for RF and for anti-CCP positivity, respectively. All analyses were carried out using STATA statistical software (Release 9.1; STATA Corporation, College Station, TX, USA). Based on the strength of the association described by Smyth and colleagues in T1D [14], our study had a 73% power to detect a similar effect in RA.

Gene expression data were not normally distributed and the differences were therefore compared using the Mann–Whitney nonparametric tests. The interquartile range represents the 25th and 75th percentiles of the distribution. All gene expression analysis was performed using GraphPad Prism Version 4 (GraphPad Software, San Diego, CA, USA).

## Results

### IFIH1 A946T is not associated with RA susceptibility or severity

Allele and genotype frequencies for IFIH1 A946T were in Hardy–Weinberg equilibrium for both RA cases and controls. The frequency of the 946C allele was 0.62 and 0.60 in the patient group and in the control group, respectively (*P *= 0.3), which was similar to that reported in the T1D study [14]. Genotypes were not significantly different, indicating that this polymorphism is not a susceptibility gene for RA (Table 1). To determine whether this marker influenced the disease phenotype, patients were stratified according to demographic and clinical features. Allele frequencies and genotypes were not significantly different after stratification by sex, age at onset, presence of RF or presence of anti-CCP (Table 1). The modified Larsen score was not significantly different in patients of the three genotypes.

**Table 1:** Genotype frequencies of the interferon induced with helicase domain 1 A946T polymorphism

	Genotype frequency		
	
	GG	AG	AA
Rheumatoid arthritis			
Patients	126 (13.7%)	446 (48.5%)	348 (37.8%)
Controls	144 (15.5%)	450 (48.4%)	335 (36.1%)
Odds ratio (95% confidence interval)	0.8 (0.6–1.1)	0.9 (0.8–1.2)	
*P *value	0.2	0.6	
Rheumatoid factor			
Positive	76 (13.1%)	281 (48.5%)	222 (38.3%)
Negative	40 (15.2%)	126 (47.9%)	97 (36.9%)
Odds ratio (95% confidence interval)	0.8 (0.5–1.3)	1.0 (0.7–1.4)	
*P *value	0.4	0.9	
Cyclic citrullinated peptide			
Positive	87 (13.1%)	328 (49.2%)	251 (37.7%)
Negative	31 (15.1%)	99 (48.3%)	75 (36.6%)
Odds ratio (95% confidence interval)	0.8 (0.5–1.4)	1.0 (0.7–1.4)	
*P *value	0.5	1.0	
Larsen score			
Median	36.0 (13.4%)	27.5 (48.3%)	27.0 (38.3%)
*P *value		0.2	

We assumed a multiplicative inheritance model by performing logistic regression. The model with the largest log-likelihood ratio was chosen as the one that best represented the mode of inheritance of the data.

### IFIH1 mRNA levels in peripheral blood mononuclear cells of RA patients and healthy controls

**Figure 1:**
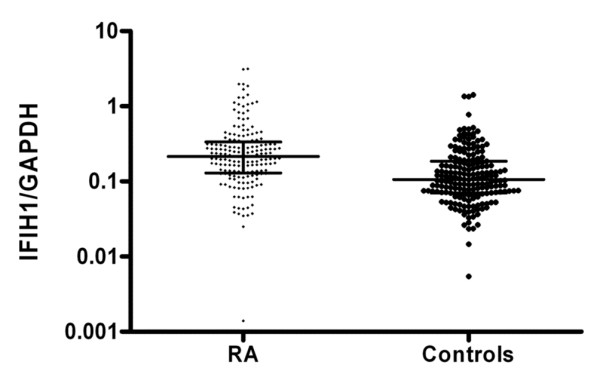
Expression of interferon induced with helicase domain 1 mRNA in peripheral blood mononuclear cells. Total RNA was extracted from whole blood of rheumatoid arthritis (RA) patients and healthy controls, and the transcript levels were measured using real-time PCR. Data expressed as the ratio of interferon induced with helicase domain 1 (IFIH1) mRNA copies to those of GAPDH. Lines, median values; bars, interquartile ranges.

Levels of IFIH1, expressed as the ratio of IFIH1 mRNA copies to those of GAPDH, were significantly higher in RA cases (0.21 ± 0.5) than in healthy controls (0.10 ± 0.2) (*P *< 0.0001) (Figure 1). We further investigated whether the IFIH1 A946T genotype was associated with mRNA levels. The IFIH1 mRNA levels in patients homozygous for the A allele (*n *= 63) and in patients heterozygous for (*n *= 79) and homozygous for the G allele (*n *= 29) were 0.20 ± 0.3, 0.23 ± 0.6 and 0.18 ± 0.3 (*P *= 0.98), respectively. Similar results were observed in healthy controls: IFIH1 mRNA levels were 0.10 ± 0.2, 0.11 ± 0.2 and 0.12 ± 0.1 (*P *= 0.7) for A homozygote controls (*n *= 76), for heterozygous controls (*n *= 85) and for G homozygote controls (*n *= 22), respectively.

## Discussion

Our results indicate that the IFIH1 A946T SNP is not associated with susceptibility to RA. Although the present study involved large populations it only had modest power (73%) to detect an effect of the magnitude reported recently in T1D, and therefore additional studies using large RA cohorts are required to fully exclude an effect from IFIH1. Genetic susceptibility to RA has been examined extensively; however, relatively few studies have investigated the role of genetic factors in RA severity. There is evidence of a significant impact on radiological damage that is independent of covariates including disease duration and RF status [16].

A recent study compared the variance in radiological hand damage in monozygotic and dizygotic twins and pairs of unrelated RA patients. After assuming a linear relationship between radiological progression and disease duration, the variation in joint destruction was highest in unrelated pairs, followed by dizygotic twins, and was smallest between monozygotic twins supporting a genetic input [17]. We did not detect a significant association of this marker with radiological damage. Our results are consistent with linkage studies in RA that have not found a genetic contribution for the region at 2q24.3 that encodes IFIH1 [18].

The genetic association of IFIH1 with T1D may be explained by the reported link with preceding viral infections, as this gene is thought to contribute to the apoptosis of virally infected cells [19]. Viral RNA is sensed via a helicase domain resulting in the activation of an N-terminal caspase recruitment domain, which activates several key downstream pathways including NF-κB and IRF3 [20]. The A946T SNP does not reside in either the helicase or CARD domains, and therefore the biological effects of this variant are unknown – although the variant does lie in a region that is highly conserved between mammals, suggesting functional importance. The role of viruses in the pathogenesis of RA has been suggested, and a polyarthritis resembling RA has been described after infection with Epstein–Barr virus, parvovirus B19, HTLV-1 and human herpes-6 or human herpes-8; however, evidence for a role of viruses in RA is circumstantial and inconclusive [21].

Although no association between this genetic variant and RA was detected, IFIH1 mRNA levels were greater in RA patients compared with healthy controls. This gene is expressed at high levels in immune such as CD4^+ ^T cells, CD8^+ ^T cells, CD19^+ ^B cells, monocytes and dendritic cells (USCS Genome Browser–GNF expression Atlas, [22]) The upregulation of IFIH1 in RA patients could be explained by the increased expression of IFNβ because IFIH1 is a highly IFNβ-inducible protein that is thought important in mediating IFNβ effects such as growth inhibition and apoptosis [23]. Immunohistochemical analysis has shown IFNβ to be highly expressed in rheumatoid synovium in fibroblast-like synoviocytes, dendritic cells and macrophages. Our finding of increased IFIH1 expression in peripheral blood leucocytes of patients suggests that the increased levels of IFIH1 may result from overexpression of this immunomodulatory cytokine [24]. An alternative explanation is that the increase is a reflection of a difference in the cellular subpopulations in the peripheral blood of RA patients and controls.

## Conclusion

We conclude from the results of the present large case–control study that there is no significant role of the A946T IFIH1 polymorphism in the genetic susceptibility to RA or with the development of more severe radiological damage. Levels of IFIH1 mRNA were approximately twofold higher in peripheral blood leucocytes of patients compared with controls, suggesting upregulation in inflammatory cytokines such as IFNβ.

## Abbreviations

CCP = cyclic citrullinated peptide; IFIH1 = interferon induced with helicase domain 1; IFN = interferon; NF = nuclear factor; PCR = polymerase chain reaction; RA = rheumatoid arthritis; RF = rheumatoid factor; SNP = single nucleotide polymorphism; T1D = type 1 diabetes.

## Competing interests

The authors declare that they have no competing interests.

## Authors' contributions

DSM and AGW conceived and designed the study. DEB, MHB, MCD, DJM and IM acquired the samples or study data. IM performed allelic discrimination and mRNA quantitation experiments as well as all data analyses. All authors were involved in the interpretation of data, and read and approved the final manuscript.
